# Rapid on-site identification of hazardous organic compounds at fire scenes using person-portable gas chromatography-mass spectrometry (GC-MS)–part 2: water sampling and analysis

**DOI:** 10.1080/20961790.2019.1662648

**Published:** 2019-11-15

**Authors:** Rylee Lam, Chris Lennard, Graham Kingsland, Paul Johnstone, Andrew Symons, Laura Wythes, Jeremy Fewtrell, David O’Brien, Val Spikmans

**Affiliations:** a School of Science and Health, Western Sydney University, Penrith, Australia;; b Fire Investigation and Research Unit, Fire & Rescue NSW, Greenacre, NSW, Australia;; c Operations Capability Directorate, Fire & Rescue NSW, Greenacre, NSW, Australia;; d Environment Protection Science Branch, Office of Environment and Heritage, Lidcombe, NSW, Australia;; e Hazardous Incidents and Environmental Health Branch, New South Wales Environment Protection Authority, Sydney, Australia;; f Strategic Capability, Fire & Rescue NSW, Greenacre, NSW, Australia

**Keywords:** Forensic sciences, portable GC-MS, water pollution, field analysis, SPME, fire, VOCs, SVOCs

## Abstract

Building and factory fires pose a great risk to human and environmental health, due to the release of hazardous by-products of combustion. These hazardous compounds can dissipate into the environment through fire water run-off, and the impact can be immediate or chronic. Current laboratory-based methods do not report hazardous compounds released from a fire scene at the time and location of the event. Reporting of results is often delayed due to the complexities and logistics of laboratory-based sampling and analysis. These delays pose a risk to the health and wellbeing of the environment and exposed community. Recent developments in person-portable instrumentation have the potential to provide rapid analysis of samples in the field. A portable gas chromatograph-mass spectrometer (GC-MS) was evaluated for the on-site analysis of water samples for the identification of hazardous organic compounds at fire scenes. The portable GC-MS was capable of detecting and identifying a range of volatile and semi-volatile organic compounds in fire water run-off, and can be used in conjunction with conventional laboratory analysis methods for a comprehensive understanding of hazardous organics released at fire scenes. Deployment of this portable instrumentation provides first responders with a rapid, on-site screening tool to appropriately manage the run-off water from firefighting activities. This ensures that environmental and human health is proactively protected.

## Introduction

Monitoring the environment for the presence of pollutants is a vital step in managing human and environmental health and safety [1]. Pollution can have long lasting [2] and detrimental effects to human health [3, 4] and/or the wellbeing of local environments [2]. Identifying the type of pollutant is critical to assess potential distribution pathways and toxic effects, and aids in appropriately managing and mitigating any damage caused [5]. Environmental monitoring of hazardous substances involves the detection and identification of extraneous materials present in environmental matrices and can be achieved through the sampling and analysis of air, water and soil [6, 7]. In addition to the protection of human and environmental health, environmental forensic investigations must also determine the source of the pollutant in order to determine criminal and/or civil liability for the assignment of clean-up costs [8].

Different forms of pollution require different levels of response. Some contamination events involve the release of a rapidly dispersing pollutant or a high concentration of pollutants that can pose significant risk to human and environmental health in a short period of time [9]. These events call for rapid, emergency response. During emergency scenarios it is vital that rapid intelligence is communicated to emergency response teams for effective and targeted risk assessment and management [10, 11]. However, traditional environmental sampling and analysis techniques do not always meet this target [8, 12]. In particular, current water monitoring procedures require extensive sample preparation steps using liquid-liquid extractions prior to lengthy analytical work [6, 7]. Due to the complexities associated with environmental sampling and analysis, results are often reported some days after the initial pollution event. It is therefore not unexpected that these results do not play a significant role in immediate, in-field emergency risk assessment and management practices. As it stands, rapid methods for in-field water sampling and analysis are limited. Any environmental protection strategies are therefore mostly reactive or precautionary.

Recent developments in person-portable instrumentation have the potential to overcome the issue of delayed laboratory results reporting [13]. Person-portable instruments afford first responders the ability to rapidly detect and identify pollutants at a contamination event, at the time and location of the incident, and can provide a more comprehensive understanding of potential contaminants at a scene throughout the duration of the event. Some available methods of in-field sampling and analysis include portable X-ray fluorescence [14, 15], Fourier transform infra-red [16] and Raman spectroscopy [17] for forensic and environmental analyses.

In the event of a large metropolitan fire, person-portable instrumentation could provide vital intelligence for the protection of human and environmental health. Fires, in particular factory fires, are responsible for the rapid release of large volumes of hazardous organic compounds as a result of off-gassing, pyrolysis and combustion of materials [6, 9]. Firefighting activities can aid the release and transport of these contaminants through the application of large volumes of water to the scene. Any water that comes into contact with the fire debris or smoke has the potential to absorb and transport products and by-products of pyrolysis and combustion. As the run-off water percolates into the surrounding soil and/or enters waterways, these pollutants are distributed into the local environment [9]. It is imperative that the detection and identification of these hazardous organics is determined during an active fire to assist first responders in community protection strategies and to minimize the release of contaminated run-off into waterways. This may include the use of bonding or absorbent materials to prevent the toxicants or contaminated water being released into the environment [18].

Through the use of person-portable instrumentation, it is possible for first responders to receive active intelligence on the release of water-borne pollutants from fire scenes [12]. This type of information gives first responders and environmental protection agencies actionable intelligence for proactive and targeted environmental and human health risk management and protection strategies. One such instrument that could meet the needs for first responders is the portable gas chromatograph-mass spectrometer (GC-MS). A portable GC-MS unit that has been previously explored at fire scenes [12, 19] is the Torion T-9. This unit is a small person-portable GC-MS that weighs approximately 15 kg and runs off on-board batteries and helium, making it truly portable for field-based analysis. The additional advantage of this particular instrument is its design for solid phase microextraction sampling (SPME), eliminating the need for the transport and use of organic solvents in the field.

The aim of this research was to develop field-based sampling and analysis methods using portable GC-MS for the rapid identification of hazardous organic compounds in water run-off generated from firefighting activities. Identification of these pollutants would aid in determining the response to firefighting run-off water to prevent environmental impact, where possible. The developed in-field methods are to be used in conjunction with laboratory-based methods to provide rapid intelligence that is currently not available. The in-field methods are not designed to replace the laboratory analysis.

## Materials and methods

A series of small-scale controlled burns consisting of different construction materials were performed. These fires were extinguished using water and the resulting run-off water was collected for analysis by a portable GC-MS. The results were examined to determine the ability of the portable GC-MS to detect, separate and identify volatile and semi-volatile organic compounds in fire water run-off. The compounds detected in the water run-off across the different materials were compiled into a library. Water samples were collected for analysis using common laboratory-based methods for comparison with the field-based results.

### Materials

A range of household and building materials, obtained from local building and hardware stores, were selected for their abundance within modern buildings. The following six construction materials were used during the experiments: particle board sheet (5 cm × 2 cm × 1 cm), melamine coated particle board (5 cm × 2 cm × 1 cm), laminated plywood sheet (5 cm × 3 cm × 2 cm), floating flooring rubber underlay (5 cm × 5 cm × 0.5 cm), pile carpet tile with a rubber backing (4 cm × 4 cm × 0.5 cm) and a rubber gym mat made from recycle tyres (5 × 5 × 1 cm). Each material was burned in triplicate, under controlled conditions, for a total of 18 test burns.

The materials were selected on the basis of ease of access and occurrence within the Australian built environment. For example, in 1999/2000, 978 000 m^3^ of particle board and 192 000 m^3^ of plywood were manufactured in Australia, where a total of 78.5% particle board and 99.4% of plywood were used for furniture, kitchens, structural (including exteriors, formwork and flooring) and building industries [20].

### Experimental set-up

The burns of the materials were conducted in a bunded metal tray to ensure that any water used to extinguish the fire was contained. The metal tray was lined with aluminium foil and replaced every burn, to prevent cross-contamination between different burns. As most construction materials require sustained heat to ignite, a small pile of paper and cardboard, lit using a match, was used to start the fire. The construction materials were suspended vertically over the burning paper and cardboard using a retort stand. Once the construction materials were fully alight for 2–3 min, the fire was extinguished using water. Additional triplicate burns were also conducted using just paper and cardboard to determine the contribution these ignition materials had on the chemical profiles.

### Sample collection and extraction method

For each burn, four 22 mL glass screw cap headspace vials with PTFE lined septa (Restek, Bellefonte, PA, USA) were prepared as indicated in Table 1. One vial was prepared for headspace sampling (HS), by adding 2 g of sodium chloride salt (ACS Reagent; Sigma Aldrich, Darmstadt, Germany). A magnetic stirrer was placed in two other vials to aid with direct immersion (DI) sampling, whilst the fourth vial remained empty. These water samples were collected from each fire for the purpose of comparison between different sampling and analysis methods.

**Table 1.** Run-off water samples collected from controlled fires. Ten mL of run-off water was placed into each vial. Each set of four vials was collected for each controlled fire, including for the fires containing only paper and cardboard.

Vial No.	Preparation	Sample extraction method	Analysis method	Location of sampling and analysis
1	Unfiltered samples added to vial containing 2 g salt	HS-SPME	Portable GC-MS	In-field
2	Sample filtered into vial with stirrer bar	DI-SPME	Portable GC-MS	In-field
3	Sample filtered into vial with stirrer bar	DI-SPME	Benchtop GC-MS	Laboratory
4	Unfiltered sample added to empty vial	Liquid-liquid extraction	Benchtop GC-MS	Laboratory

HS-SPME: headspace sampling-solid phase microextraction sampling; GC-MS: gas chromatography-mass spectrometry; DI-SPME: direct immersion sampling-solid phase microextraction sampling.

A water sample was collected from the residual run-off water in the bottom of the tray. The water was poured into a clean 100 mL glass beaker. The water was mixed prior to transferring 10 mL aliquots into the four headspace vials using a syringe. For the samples to be used with DI-SPME sampling, the water was filtered using a 0.45 µm nylon filter (LabServ; Thermo Fisher, Waltham, MA, USA) (Table 1). Samples were also collected from run-off water of paper and cardboard fires.

### SPME extraction method

Field sampling on Vials 1 and 2 and laboratory-based sampling on Vial 3 were conducted using a Custodion SPME needle with a polydimethylsiloxane/divinylbenzene (PDMS/DVB) fibre (Perkin Elmer Inc., Waltham, MA, USA). The water was filtered using a 0.45 µm filter prior to adding to Vials 2 and 3 (Table 1). Filtering the samples prior to DI-SPME was as a necessary precaution against damage to the SPME fibres as a result of particulate matter blocking the sorbent phase. Conventional laboratory-based extraction methods, as performed on Vial 4, took the entire sample phase (liquid and particulate matter) and, as such, the filtration step was not conducted on these water samples. Whilst this may appear to be a disparate treatment of samples, it is necessary to compare the field-based sampling and analysis methods to the laboratory-based sampling and analysis methods as currently conducted operationally.

Vials 1, 2 and 3 were shaken vigorously for 1 min prior to sampling. An HS-SPME sample was collected from Vial 1 by piercing the septa of the vial with the SPME needle and exposing the sorbent fibre to the headspace within the vial for 10 min. Salt was used to assist in the dissociation of compounds from the liquid phase into the headspace of the vial for more efficient extraction [21–23]. Once the fibre was retracted from the headspace and the needle removed from the vial, the SPME fibre was ready for analysis.

A DI-SPME sample was taken from Vials 2 and 3 using a similar approach. The SPME needle was inserted into the vial through the septum. The fibre was immersed into the liquid phase of the run-off water, which was stirred continuously during the 10 min exposure, using a portable, battery-operated magnetic stirrer (Labtek, Brendale, Australia). Continuous stirring was required to increase the extraction efficiency of the SPME fibre through increasing the volume of sample that came into contact with the sorbent [21, 23].

The HS- and DI-SPME samples collected from Vials 1 and 2 were analyzed on-site using a Torion T-9 portable GC-MS (Perkin Elmer Inc.) as discussed below. The DI-SPME sample from Vial 3 was analyzed on a benchtop GC-MS in the laboratory as described below.

### Liquid-liquid extraction (LLE) method

Vial 4 water samples were extracted using conventional extraction procedures [24, 25]. LLEs and subsequent GC-MS analysis was conducted on combined water samples collected from triplicate burns of the same material. Vial 4 was taken from each burn of the same material and combined prior to extraction as a single representative water sample for that material. Due to the small volume of water required for extinguishment of these test burns, a full extraction volume of 100 mL required for the LLE method could not be collected from each individual burn. Thus, water samples collected from the triplicate burns were combined to increase the extraction volume, and therefore improve compound detection. As the final results were to be combined to form a summed total of compounds identified, no loss of data occurred by combining the three replicate water samples. The final volume was made up to 100 mL using ultrapure water (>18 MΩ.cm; MilliporeSigma, Burlington, MA, USA). The combined sample was placed in a dichloromethane (DCM) (EMSURE^®^ grade, Merck, Darmstadt, Germany) rinsed 250 mL separation funnel. Surrogate and quality control standards were then added. Next, 25 mL of DCM was added to each sample in the separation funnels and shaken for 2 min, prior to letting it rest for 15 min. The lower organic layer was then extracted into TurboVap funnels, that were prepared by placing a wad of glass wool (silanized; Sigma-Aldrich, Saint Louis, MO, USA) in the tip of the funnel followed by a layer of anhydrous sodium sulphate (10–60 mesh, analytical reagent grade; Chem-Supply, Gillman, Australia). The DCM extraction was repeated a further two more times for a total of three extractions. The TurboVap funnel was rinsed with a small volume of DCM and placed in a TurboVap^®^ II Concentration Workstation (Biotage AB, Uppsala, Sweden) at 36 °C under nitrogen for 20–30 min. Once the sample was concentrated down to approximately 5 mL, the concentrated sample was pipetted into a clean 10 mL glass vial. The funnel was rinsed with a small amount of DCM, and the rinse was added to the glass vial. The vial was then placed in a Thermo Scientific Reacti-Therm III #TS-18823 Heating/Stirring Module (Thermo Fisher Scientific) at approximately 36 °C under nitrogen and further concentrated down to <1 mL. The final sample concentrate was transferred to a 1 mL glass GC-MS vial with screw-cap lid and made up to exactly 1 mL with DCM using a syringe. Internal standards (1,4-dichlorobenzene-d4, acenaphthalene-d10 and chrysene-d12) (Sigma-Aldrich) were added to the final extract to enable surrogate recovery calculations.

### Analysis methods

#### Torion T-9 portable GC-MS

Water samples in Vials 1 and 2 were analyzed immediately after sample collection and preparation using a Torion T-9 portable GC-MS. The injection port of the portable GC-MS was set to desorb the SPME fibre for 5 s at 270 °C, with an initial split ratio of 10:1. Ten seconds after injection the split ratio was increased to 50:1 for a further 20 s. The split line was closed 30 s after injection. The GC column used was a 5 m MXT-5 Crossbond diphenyl dimethyl polysiloxane (0.1 mm ID × 0.4 µm film thickness). Column temperature was held at 50 °C for 10 s then increased at 2 °C/s to a final temperature of 270 °C. The final temperature was held for 60 s for a total run time of 180 s. The sample was transferred to an ion trap mass spectrometer at 250 °C. Detection was conducted over a mass range of 43–500 u.

The methods loaded on the portable GC-MS also include an automated data processing step that compares compound spectral data to an on-board library to produce a list of compound identifications. The on-board target library was generated by the manufacturer. Due to the limited size of the on-board library, not all compounds could be automatically identified. Additional compounds were identified in the chromatograms with the use of deconvolution software (Chromion version 1.2.0.8; Perkin Elmer Inc.) and manual matching of spectral data to the NIST mass spectral library [26]. Any compounds that were repeatedly identified using NIST searching were added to the on-board library for ease of future identification.

#### Shimadzu GC-MS

A comparison study between the portable GC-MS and benchtop GC-MS systems was conducted using the DI-SPME sampling method. The Vial 3 water samples were analyzed, after DI-SPME extraction, using a Shimadzu QP2010 Ultra benchtop GC-MS (Shimadzu Scientific Instruments Pty. Ltd., Tokyo, Japan). The method parameters were set using GCMS Real Time Analysis Software (Shimadzu GCMSsolution Version 4.20) and involved inserting the SPME needle into the GC-MS and exposing the fibre in the injection port to a 1-min desorption time. The injection port was set for a splitless injection at 270 °C. A 30 m, 0.25 mm ID, 0.25 µm film thickness Restek Rtx 5 MS column with a Crossbond 5% diphenyl, 95% dimethyl polysiloxane stationary phase was used. The column flow rate was set to 1.05 mL/min with the following temperature program: initial 40 °C hold for 2 min, then a 10 °C/min ramp to 300 °C, which was held for a further 10 min for a total run time of 38 min. The GC to MS interface temperature was set to 200 °C. The MS ion source temperature was 250 °C and the MS was set to scan over a mass range of 35–500 u.

The chromatograms were processed to identify compounds present using GCMS Post Analysis Software (Shimadzu GCMSsolution Version 4.20) and the Shimadzu Mass Spectrum NIST library 2011 [27]. The list of identifications was compared to the list of compound identifications made in the field using the Torion T-9 Portable GC-MS, to determine any similarities and/or differences obtained in the results from the conventional benchtop and person-portable instrumentation.

#### Conventional laboratory analysis

The samples prepared by LLE were analyzed on an HP 5973 Series GC-MS System (Hewlett Packard, Palo Alto, CA, USA). The GCMS parameters were set using Environmental ChemStation (MSD ChemStation version E.02.02.1431; Agilent Technologies Inc., Santa Clara, CA, USA). A total volume of 2 µL of extract was injected into the GCMS. The injection temperature was set to 250 °C with a pulsed splitless injection time of 1 min. A 30 m, 0.25 mm ID, 0.25 µm film thickness Agilent J&W DB-5MS Ultra Inert column with a 5% diphenyl, 95% dimethyl polysiloxane stationary phase was used. The column flow rate was set to 1 mL/min with the following temperature program: initial 40 °C hold for 2 min then a 10 °C/min ramp to 300 °C, which was held for 12 min for a total run time of 40 min. The GC to MS interface temperature was 200 °C. The MS ion source temperature was 230 °C and the MS was set to scan over a mass range of 50–450 u with a 6.5-min solvent delay.

The chromatograms were processed to identify target compounds present in the samples using the Environmental ChemStation software. These results were then compared to results obtained using in-field methods. Non-target compounds were identified using the NIST 2008 reference mass spectral library.

## Results and discussion

### In-field sampling and analysis

**Figure 1.**
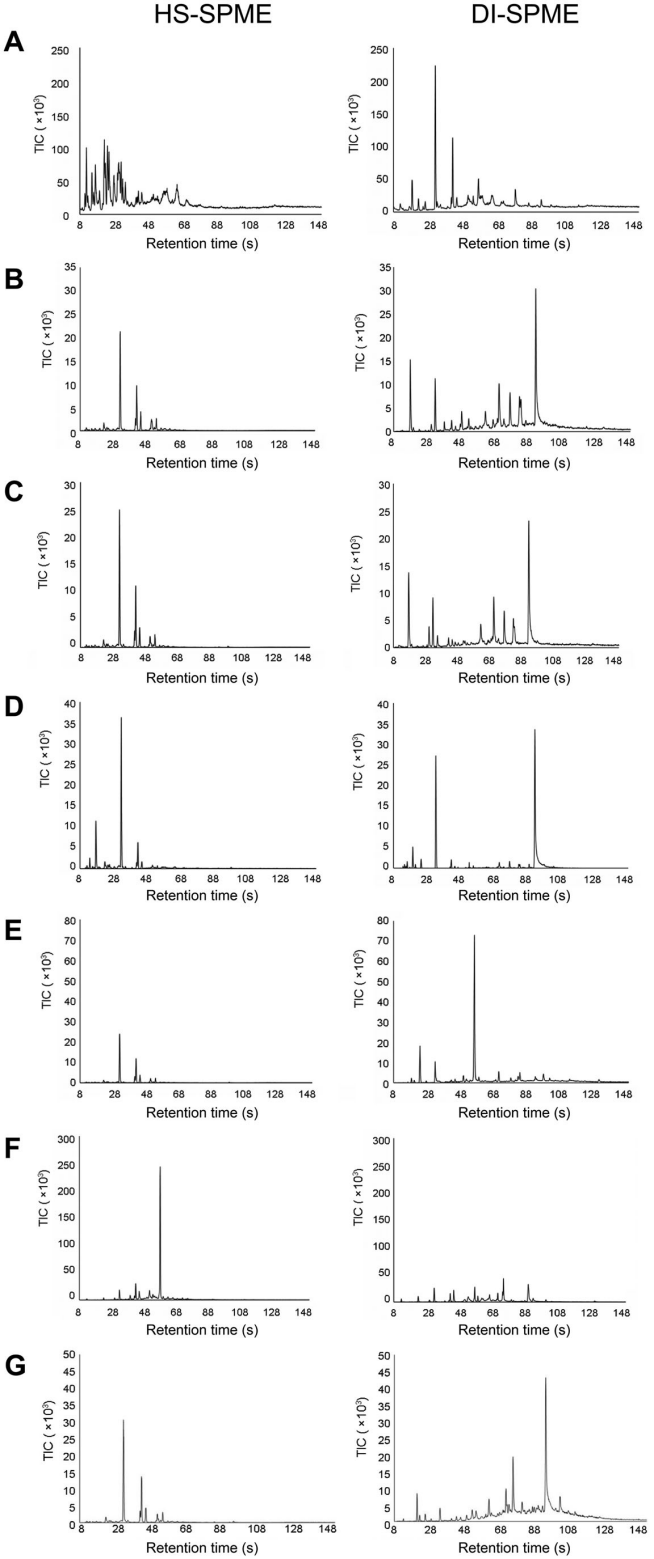
Representative portable gas chromatography-mass spectrometry (GC-MS) chromatograms of water run-off samples collected after extinguishment of fires consisting of (A) background paper and cardboard (max temp. ∼600 °C), (B) particle board (max temp. ∼500 °C), (C) melamine coated particle board (max temp. ∼500 °C), (D) laminated wood (max temp. ∼600 °C), (E) carpet (max temp. ∼300 °C), (F) rubber (max temp. ∼550 °C) and (G) underlay (max temp. ∼650 °C). Samples were extracted using both headspace sampling-solid phase microextraction sampling (HS-SPME) and direct immersion sampling-solid phase microextraction sampling (DI-SPME). Chromatograms have been adjusted to the same scale for direct comparison between the two extraction methods.

To determine if the field-based HS-SPME and DI-SPME sampling methods combined with the field-based portable GC-MS analysis were capable of rapid on-site water sampling and analysis, the methods were assessed for their ability to detect and identify a range of volatile organic compounds (VOCs) and semi-volatile organic compounds (SVOCs) in water run-off samples. The results of these analyses are shown in Figure 1.

The HS-SPME method primarily extracted light VOCs that eluted before 70 s in the chromatogram, whereas DI-SPME was able to extract heavier VOCs and SVOCs. Whilst the DI-SPME method extracted most of the compounds also detected by the HS-SPME method, the signal intensity of the lighter VOCs was higher in the HS-SPME extraction than in the DI-SPME extraction of the same sample. Conversely, heavier SVOCs were present in greater intensities in the DI-SPME extraction and were absent from the HS-SPME results.

Table 2 provides an overview of the range of compounds that were detected in the run-off water from the different material burns. Compounds were considered identified if, based on similarity scores followed by a visual inspection of the mass spectra, a match with either the standard on-board library or the NIST database could be made. The portable GC-MS generates mass spectra using an ion trap and the NIST database is mainly generated using quadrupole mass spectrometers. Identifications using similarity thresholds alone is therefore not suitable and visual identifications were required. In addition, a compound was not considered identified unless the same compound identification was obtained from multiple independent analyses.

**Table 2.** Target compound list for in-field fire run-off water sampling and analysis.

Compound name	Paper/cardboard		PB		Mel		LW		Carpet		Rubber		Underlay	
	HS	DI	HS	DI	HS	DI	HS	DI	HS	DI	HS	DI	HS	DI
Trimethylamine				✔		✔		✔						
Pentane [28]							✔							
Methylbutadiene isomer [29]											✔			
Methylbutane isomer [30, 31]	✔	✔	✔				✔	✔	✔				✔	
Dimethylbutane isomer	✔	✔	✔		✔			✔				✔		
Methylene chloride							✔	✔						
Pentenyne												✔		
Methylbutadiene isomer											✔	✔		
Methybutene isomer [31]		✔	✔					✔	✔			✔		
Methylpentane isomer [32]	✔		✔		✔		✔	✔	✔				✔	
Methylpentane isomer	✔	✔	✔		✔		✔	✔	✔				✔	✔
Hexane [31–33]	✔	✔					✔					✔		
Dimethyloctane isomer				✔		✔		✔		✔				✔
Methylfuran isomer						✔		✔						
Unknown 1		✔		✔		✔	✔	✔		✔		✔	✔	✔
Hexane			✔		✔		✔		✔				✔	
Methyloctene isomer	✔				✔				✔				✔	
Methylpentanol isomer	✔					✔	✔	✔						✔
Tetrahydrofuran			✔											
Butanol [29]			✔				✔	✔						
Benzene [28–38]	✔	✔	✔	✔	✔	✔	✔	✔	✔	✔	✔	✔	✔	✔
Dimethylcyclopentane isomer	✔								✔					
Chloromethylbutane isomer	✔	✔	✔											
Trimethylpentane isomer [32]	✔	✔	✔		✔		✔	✔	✔				✔	
Ethyldimethylpentane isomer	✔	✔			✔		✔	✔	✔				✔	
Heptane [31]	✔	✔	✔		✔		✔	✔	✔				✔	
Dimethylfuran isomer [29]	✔			✔		✔								
Trimethylpentane isomer	✔						✔		✔					
Methylpentanone isomer											✔			
Methylpyrrole isomer				✔		✔								
Trimethylpentane isomer	✔				✔		✔		✔					
Dimethylbutane isomer	✔		✔											
Methylhexanal isomer	✔						✔		✔					
Trimethylpentene isomer	✔				✔		✔						✔	
Toluene [28, 29, 31–40]	✔	✔	✔	✔	✔	✔	✔	✔	✔	✔	✔	✔	✔	✔
Butanone [29]	✔		✔		✔		✔		✔				✔	
Hexanal [29]						✔								
Unknown 2						✔								
Furfural [29, 35, 41, 42]		✔		✔		✔				✔		✔		
Unknown 3											✔			
Ethylbenzene [29, 35, 36, 38, 40]	✔		✔	✔	✔	✔	✔	✔	✔		✔	✔	✔	✔
Xylene isomer [28–31, 36, 40]		✔	✔	✔	✔	✔	✔	✔	✔	✔	✔	✔	✔	✔
Phenylethyne [29, 40]											✔	✔		
Styrene [29, 32, 34, 35, 37, 40]		✔		✔		✔	✔	✔			✔	✔		✔
Xylene isomer	✔	✔	✔	✔	✔	✔	✔	✔	✔		✔	✔	✔	
Cyclohexanone [42]												✔		
Unknown 4				✔		✔								✔
ɑ-Pinene [29]				✔										
Ethylhexanal isomer										✔				
Trimethylbenzene isomer [36]		✔	✔	✔	✔		✔	✔	✔		✔		✔	✔
Unknown 5											✔			
Trimethylbenzene isomer			✔	✔	✔				✔				✔	
Methylfurancarboxyaldehyde isomer [40]										✔				
Unknown 6				✔										
Benzaldehyde [29, 40]		✔		✔		✔		✔		✔				
Camphene [29]											✔	✔		
Propylbenzene isomer [40]			✔	✔	✔				✔				✔	
Phenol [29, 34, 35, 37]												✔		
Aniline											✔	✔		
Benzonitrile [29, 38, 40]										✔				✔
Trimethylbenzene isomer		✔	✔		✔		✔	✔	✔				✔	✔
Unknown 7											✔			
Unknown 8								✔						
Methylethylheptane isomer										✔				
Trimethylbenzene isomer					✔				✔					
Limonene [29, 36]			✔											
Dimethylpyrrole isomer								✔						
β-Pinene		✔						✔				✔		
Unknown 9											✔			
Methylphenol isomer										✔				
Indene [29, 36, 37, 40]										✔		✔	✔	
Unknown 10											✔			
Acetophenone [29, 40]										✔				
Methylbenzaldehyde isomer														✔
Methylphenol isomer										✔		✔		
Methylbenzaldehyde isomer													✔	✔
Unknown 11											✔			
Methoxyphenol isomer [29, 40, 42]				✔		✔				✔				
Dimethylphenol isomer				✔						✔				
Methylindene isomer										✔				
Dimethylphenol isomer				✔										
Methoxymethylbenzene isomer				✔										
Unknown 12												✔		
Unknown 13														✔
ɑ-ɑ-Trimethylcyclohexene-methanol isomer						✔								
Methoxymethylphenol isomer				✔		✔	✔	✔		✔				
Naphthalene [28, 29, 32–34, 36, 39, 40, 43]		✔		✔				✔		✔	✔	✔	✔	✔
Unknown 14														✔
Dimethoxytoluene isomer				✔			✔	✔						
Unknown 15												✔		
Benzothiazole											✔	✔		✔
o-(Methoxybenzoyl)-o'-(chlorobenzoyl)-benzenediol isomer												✔		
Ethylmethoxyphenol isomer [35]				✔		✔	✔	✔		✔				
Unknown 16										✔				
Methylbenzothiazole isomer														✔
Methylnaphthalene isomer [35, 36, 40]												✔	✔	✔
Hydroxylmethylacetophenone isomer										✔				
Methylnaphthalene isomer												✔		✔
Unknown 17										✔				
Eugenol [41, 42]				✔		✔	✔	✔						
Propenylmethoxyphenol isomer [42]				✔				✔		✔				
Unknown 18										✔				
Propylmethoxyphenol isomer [35]						✔	✔	✔						
Tetradecane [40]				✔										
Eugenol [41, 42]				✔										
Eugenol						✔	✔	✔		✔				
Dihydrotrimethylquinoline isomer												✔		
Unknown 19												✔		
Bis(dimethylethyl)phenol isomer		✔	✔	✔	✔	✔	✔	✔				✔	✔	✔
Unknown 20										✔				
Unknown 21										✔				
(Methylthio)-benzothiazole												✔		
Unknown 22														✔
Benzophenone										✔				
Bis(methylene)-benzamine isomer												✔		
Butyl citrate										✔				✔

PB: results obtained for particle board; Mel: results for melamine coated particle board; LW: laminated wood results; ✔: compound detected. HS: headspace; DI: direct immersion. Where a compound name has been underlined, these indicate USEPA priority pollutants [44]. Unknown compounds indicate that a clearly defined peak was present within the sample that could not be matched to either the on-board or NIST library. Citations indicate that a similar compound was previously identified as by-products of pyrolysis and combustion within the existing literature. Only the first isomer of each compound was referenced.

If the match to the library was an isomeric compound, this was indicated in Table 2 without identifying the specific isomer. In the case of alkanes, these are difficult to confidently identify without the use of alkane standards, therefore, the closest isomer or base structure was identified, to provide an overview of the range of aliphatic compounds present in the samples. As a result, the same isomer or compound may have been listed on more than one occasion in Table 2, but this indicates that multiple arrangements of that compound are present within the dataset. Ideally, the compound identifications are checked using standards, however, this was not practical based on the large number of compounds present in the samples.

Any high intensity peaks that could not be matched to either database were included as unknowns. It was important to consider these high signal intensity unknown compounds, as they have the potential to be hazardous. The databases used to make compound identifications were not developed specifically for fire water run-off, and as such, compounds could be present in the samples that have not been previously recorded. With further development and analysis, these libraries can be tailored to ensure compound identifications in future casework samples can be made with greater certainty.

Benzene, toluene, ethylbenzene and xylenes (BTEX) were present for all materials that were burned (Table 2), including paper and cardboard, whilst trimethylbenzene (TMB) was present for most materials. Because paper and cardboard are present in the burns of the other materials, this research cannot prove that all the construction materials generate these compounds. However, based on past research (refer to references provided in Table 2), it is highly likely that BTEX and TMB are present in water run-off when these materials are burned. In addition to BTEX and TMB, a range of light aliphatic compounds were observed in many of the samples. These compounds were also observed during pyrolysis and combustion experiments discussed in the literature (refer to literature references in Table 2).

While it is difficult to distinguish between the paper and cardboard or the construction materials as the precise source of some of the detected compounds, some differences between the chemical profiles of the different materials can be observed. During the construction material burns, additional compounds are detected that are not present when only paper and cardboard were burnt. Therefore, the burning of different materials resulted in a varied compound profile, where additional compounds were detected, including branched phenols and methylbenzaldehydes.

The wood-based materials (particle board, melamine coated particle board and laminated wood) are similar to each other for a range of compounds, but the overall compound profile is different. The same rationale applies to the rubber-based materials of carpet (which contained a rubber backing), rubber and flooring underlay. The rubber-based materials showed different profiles to the wood-based materials. The combustion of rubber-based materials generated more nitrated compounds and larger PAHs such as methylnaphthalene than the wood-based materials.

The range of compounds detected using the in-field water sampling and analysis method indicates that there are a large number of contaminants present in fire run-off water, especially considering the small amount of debris generated by the experiments. Any water run-off that has come into contact with fire and/or fire debris has the potential to leech or run off site and contaminate the surrounding environment. Some of the compounds detected are priority pollutants monitored by USEPA methods, and these have been highlighted in Table 2 [44]. Traditionally, these priority pollutants are monitored in fire water run-off using laboratory-based methods. Therefore, it is important to note that the preliminary detection of these compounds within the field is a significant advantage for environmental protection strategies.

Many of the detected compounds have previously been identified in the literature (refer to references provided in Table 2). Much of the literature involves bench- or lab-scale combustion experiments where analysis is conducted using laboratory benchtop instrumentation. The field-based methods were able to detect similar compounds as detected in the literature, indicating that the developed field-based method was able to accurately detect and identify pyrolysis and combustion compounds in water run-off. This provides an external validation of the in-field compound identifications. Nevertheless, compounds that, as far as the authors are aware, have not been discussed in the literature were also detected. This provides additional information on the hazardous organic compounds that could be present and may be released from a fire scene.

The dataset provided contributes to the overall knowledge base and provides for the basis of a target library that is fire specific. A fire specific compound library will allow for enhanced compound identifications in the future and will develop over time as more fire scenes are attended.

### Comparison of the portable GC-MS to benchtop instrumentation

To determine the suitability of the in-field analysis method, the results obtained on the portable GC-MS were compared to those obtained on a benchtop GC-MS, to determine if a similar range of compounds could be identified by both methods. The same DI-SPME sampling technique was used to ensure any variability in results was related to the analysis technique and not the sampling. DI-SPME was able to extract a broader range of compounds than HS-SPME and many of the compounds extracted by the HS-SPME were also extracted by using DI-SPME. Although these compounds were present at lower concentrations in the DI-SPME, they could still be observed.

**Figure 2.**
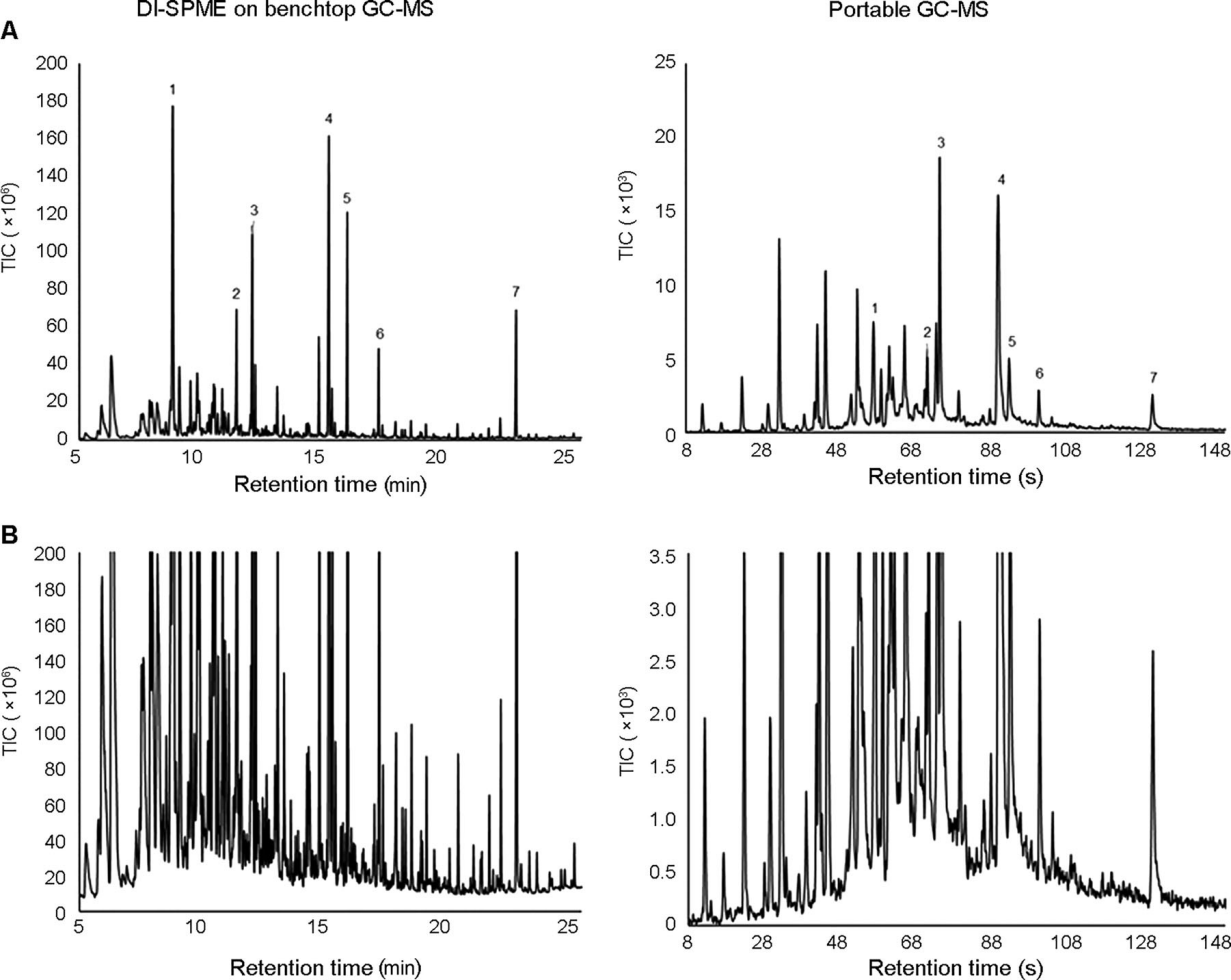
Representative benchtop gas chromatography-mass spectrometry (GC-MS) chromatogram compared to a portable GC-MS chromatogram of water run-off from a controlled burn of rubber after extraction using direct immersion sampling-solid phase microextraction sampling (DI-SPME), where (A) is a complete overview of the chromatogram and (B) is an expanded section of the baseline of the same result. The major peaks (1–7) present within the benchtop GC-MS results were also detected and identified on the portable GC-MS (see also Table 2); 1) Unknown 11, 2) naphthalene, 3) benzothaizole, 4) dihydrotrimethylquinoline, 5) bis(dimethylethyl)phenol, 6) (methylthio)-benzothiazole, 7) bis(methylene)-benzenamine.

Overall, the chemical profiles of the run-off water on the benchtop GC-MS were similar to those produced by the portable GC-MS. The results were not expected to be identical as the portable GC-MS results were obtained in the field, whereas the benchtop GC-MS results, including DI-SPME sampling, were obtained under controlled laboratory conditions. Nevertheless, all major peaks detected using the field-based method were also detected using benchtop GC-MS for all the materials burned. Figure 2 shows a representative benchtop GC-MS chromatogram of a DI-SPME extraction of a water sample obtained after extinguishment of a rubber fire. In this example, there are seven major peaks (intensity above 4.5 × 10^7^) present within the profile. These major peaks were also detected in the field using the portable GC-MS (Figure 2).

Upon closer inspection of the baseline (Figure 2(B)), a large number of additional low-level compounds can be observed. Although the portable GC-MS was able to detect many of these low-level compounds, it was not able to detect as many low-level compounds as the benchtop instrument. The greater separation capacity of the benchtop instrument is likely the reason for this.

Whilst it is clear from these results that the SPME sampling method is capable of extracting a large number of VOCs and SVOCs from the run-off water, some of these low-intensity compounds are likely lost in the background on the portable GC-MS. Due to the fast ramp rate and short column length, it is not unexpected that less trace compounds can be resolved or deconvoluted from the chromatogram compared to conventional GC analyses. Despite this result, it can be argued that it is the high intensity compounds that are most likely to be of major concern to first responders during an emergency response scenario. Compounds that are present at higher concentrations are more pressing as immediate threats to local human and environmental health. The detection and identification of these compounds becomes a priority over low-intensity compounds.

The purpose of the field-based methods is not to replace existing confirmatory analysis, but to provide a screening tool that can be used at fire scenes for proactive protection of people and the environment. If any hazardous organics are detected in run-off water at a fire scene, the sample would also be sent to the laboratory for confirmatory analysis. During this stage, these small and low-level compounds would be detected and identified, resulting in no loss of data. However, the field-based methods have provided sufficient information to classify the fire water run-off as being hazardous to the environment and potentially human health, and this on-site intelligence, obtained whilst the firefighting is occurring, can be used to initiate an immediate response to risk manage the water run-off. The fact that the field-based methods detected the presence of the major compounds, does suggest that many lower-level compounds are also present and the water run-off should be treated as such until confirmatory information is obtained from the laboratory analysis.

### Benchmarking against conventional laboratory sampling and analysis

The in-field sampling and analysis results were also compared against results obtained using traditional laboratory-based extraction and analysis methods. The purpose of this evaluation was to determine whether or not laboratory-based extraction and analysis techniques would provide a similar or different compound profile as generated using the field-based SPME extraction and portable GC-MS analysis. The results obtained by HS-SPME cannot be directly compared to a laboratory extraction method. The compounds detectable by HS-SPME are highly volatile, light-weight compounds, some of which are typically lost during the solvent evaporation step of the liquid extraction process. Therefore, only DI-SPME results were compared against the laboratory-based results. As discussed previously, the majority of the compounds detected in HS-SPME sampling are also observed using DI-SPME and hence most of these compounds are included in the comparison regardless.

**Figure 3.**
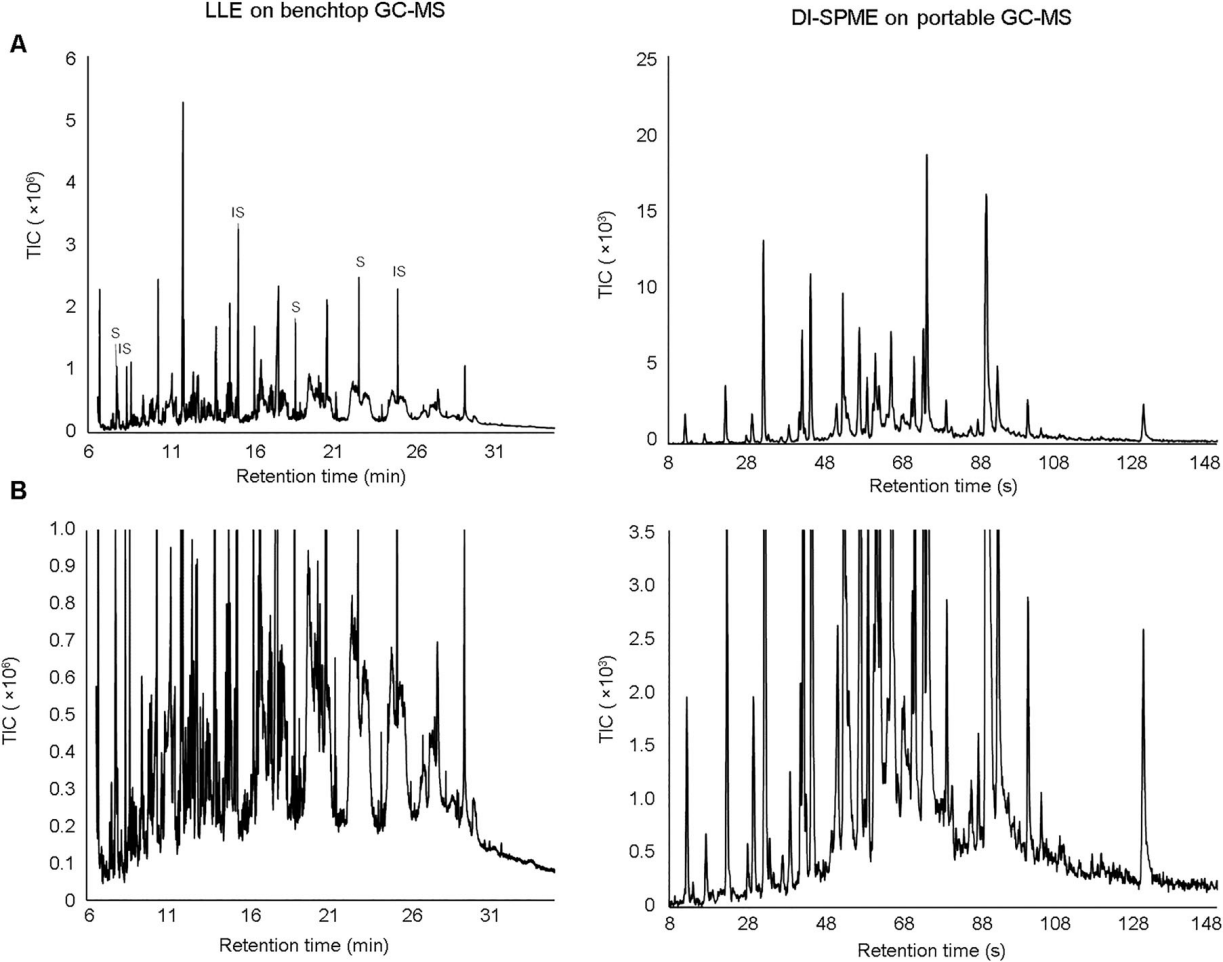
Representative benchtop gas chromatography-mass spectrometry (GC-MS) chromatogram of run-off water from a controlled burn of rubber after liquid-liquid extraction (LLE) compared to a portable GC-MS chromatogram of water run-off from a controlled burn of rubber after extraction using direct immersion sampling-solid phase microextraction sampling (DI-SPME), where (A) is a complete overview of the chromatogram and (B) is an expanded section of the same result. Surrogate compounds and internal standards in the extracted sample are denoted with “S” and “IS”, respectively.

Initial comparisons between the chromatograms generated by the portable GC-MS and the benchtop GC-MS methods show that there are a greater number of peaks present in the laboratory-based method (note that the laboratory-based results contain a range of peaks for internal standards and surrogates) (Figure 3). As explained previously, this is not an unexpected result due to the increased ability of benchtop instrumentation to resolve a large number of compounds in a complex sample through the use of a longer column combined with a longer analysis time.

Of the total number of peaks present in the samples analyzed by the laboratory-based method, only a small number (from two to five) were target compounds as set by the method. Unless a library search was performed on the other peaks, the laboratory method would not report many of the compounds detected. A NIST library scan was therefore conducted to identify unknown compounds present. The integration parameters were set to detect peaks with a minimum area count of 1 500, up to a maximum of 100 additional peaks. Due to the large volume of data that were generated using this method, only the major peaks present in the water samples from each burned material are reported in Table 3. Due to the efficiency of the extraction method and the increased sensitivity of benchtop instrumentation in comparison to the portable GC-MS, any low-intensity peaks identified using this method would not be detected using the less-sensitive field-based method. Further to this, it would also be expected that the major peaks detected using the extraction method would therefore be present within the field-based sampling and analysis results. On the other hand, all peaks detected and identified using the field-based method are expected to be present in the laboratory-based results. However, by comparing the results obtained using the field-based method (Table 2) to those obtained using the laboratory-based method (Table 3), it can be observed that although some of the compounds detected by the laboratory-based method were also detected by the field-based method, many were not.

**Table 3.** Compounds detected and identified using conventional laboratory-based sampling and analysis of fire water run-off.

Compound name	PB	Mel	LW	Carpet	Rubber	Underlay
*2(5H)-Furanone*				✔		✔
*1,2,3,3,4-pentamethylcyclopentene*	✔	✔			✔	
*Benzaldehyde*				✔		✔
*1,2,3,4,5-pentamethylcyclopentene*			✔			
Phenol	✔	✔	✔	✔	✔	✔
*2-hydroxy-3-methyl-2-cyclopenten-1-one*				✔		✔
*4-Methyl-5H-furan-2-one*				✔		✔
2-Methylphenol	✔		✔	✔	✔	✔
3 + 4-Methylphenol	✔	✔	✔	✔	✔	✔
*Methyl 2-furoate*				✔		✔
*2-methoxyphenol*	✔	✔	✔			
*Maltol*				✔		
*Benzoic acid* [29]				✔		✔
*2-methoxy-4-methylphenol*				✔		
*Dimethyl ester pentanedioic acid*					✔	
2,4-Dimethylphenol			✔	✔		
*5-(hydroxymethyl)-2-furancarboxaldehyde*	✔			✔		✔
Naphthalene					✔	
*Dimethyl ester hexanedioic acid*					✔	
*Vanillin* [41, 42]	✔			✔		
*N-phenyl-acetamide*					✔	
*2,5-bis(1,1-dimethylethyl)-phenol*						✔
*1,2-dihydro-2,2,4-trimethyl-quinoline*					✔	
*2,7-dichloro-naphthalene*	✔					
*2,5-dimethylbenzenebutanoic acid*			✔			
*2(3H)-Benzothiazolone*					✔	
*1-(4-hydroxy-3,5-dimethoxyphenyl)-ethanone*			✔			
*3-(4-hydroxy-3-methooxyphenyl)-2-propenal*			✔			
*2-Mercaptobenzothiazole*					✔	
*Octadecanoic acid*				✔		
*Butyl citrate*				✔		

PB: results obtained for particle board; Mel: results for melamine coated particle board; LW: laminated wood results; ✔: compound detected; ✔: similar peak also detected in field-based results. Citations refer to compounds previously identified as by-products of pyrolysis and combustion in the existing literature. Compounds in italics indicate library matched identifications. All other compounds are method specific target compounds.

There are several reasons that may result in a disparity between the compound identifications made by the laboratory and field methods.

The field-based method allows for the detection and identification of light VOCs. Small molecular weight organic compounds such as trimethylamine can be extracted using the field-based DI-SPME water sampling method. Light-weight compounds are typically lost during the solvent evaporation step in the LLE process and hence are not detected in the laboratory-based analyses. Therefore, the field-based method is better suited towards the detection and identification of these volatile compounds. As such, the field-based method can inherently detect and identify a wider range of organic compounds.

On the other hand, the reliance on SPME extraction during field sampling may limit the types of compounds that are detected. The field-based extraction is reliant on the sorbent phase of the SPME fibre, whereby organic compounds compatible with the sorbent phase(s) are adsorbed onto the sorbent’s surface. Compounds that are not compatible with the sorbent are not extracted from the sample matrix. In addition, any compound that might have a weak interaction with the sorbent phase could be displaced by a compound with a stronger interaction. If there are a range of compounds present within the run-off water sample that are incompatible with the sorbent phase, these will not be detected. The sorbent phase selected during these tests was a generic fibre that targets a broad range of compound chemistries [45] and acts as a general screen of the VOC and SVOCs present. Nevertheless, the SPME sampling method is not an all-encompassing screen of every compound present in the sample. On the other hand, LLE techniques rely on the partitioning of analytes from the water phase to the solvent phase; where the selected solvent determines what compounds are extracted. The range of compounds extracted by the solvent is not necessarily the same range of compounds that interact with the SPME sorbent. Any differences in the working range between sorbent and solvent selection could account for the differences between the compounds detected using the field method and those detected using the laboratory-based method.

Further observations can be made from the comparison of the laboratory- and field-based water sampling and analysis methods. The results obtained during this project suggest that the laboratory- and field-based sampling and analysis methods are complementary, and do not necessarily target the same range of compounds. The wide range of compounds that could be detected and identified using the field-based method indicated that the developed method is suitable for the preliminary identification of hazardous organic compounds in water run-off from a fire. It is true that further confirmation of compound identifications might be necessary but, as a screening tool, the methods are suitable. These preliminary results could then be used to instigate initial, precautionary environmental and human health protection measures, if necessary. Traditional laboratory-based analysis can then be used to complement the field-based results to provide for a more comprehensive overview of the chemical composition of water samples. Upon confirmatory laboratory analysis, the environmental and human health protection measures can be refined to align with the additional compounds identified during the laboratory analysis. Therefore, the incorporation of both methods into the response protocol could prove advantageous.

### Implementation at fire scenes

The results have demonstrated that SPME can be used for rapid and effective extraction of VOCs and SVOCs from water samples. In this regard, it was demonstrated during controlled field testing that a sample turnaround of four samples could be achieved in 1 h. This is a marked improvement on the traditional extraction time of 2 h per sample, plus an additional 1 h analysis time. Although the field-based method does not replace the laboratory-based analysis, it is significantly faster at providing intelligence for emergency response. SPME sampling is an easy-to-learn technique that requires minimal prior knowledge of extraction and chromatographic theory. Training on the use of SPME is primarily focussed on correct sampling and injection techniques in order to avoid damage to the SPME fibres.

There are two ways that the field-based water sampling and analysis methods can be implemented within an operational protocol. Either the firefighters collect, sample and analyze the water run-off themselves, or the firefighters collect the water run-off into pre-prepared vials and hand them to an analyst in the cold/safe zone of the scene for subsequent sampling and analysis. The first option requires additional firefighter training on the use of SPME and the portable GC-MS. The conscious effort required by the user pertains only to avoid damaging the SPME fibre and to hold the needle in place for the designated sampling time. The injection method is simple and made easier by the on-screen visual prompts provided by the Tridion-9 portable GC-MS. Therefore, any additional training and instruction on the correct use of the SPME devices is minimal. On the other hand, no further training is required if the firefighters were to simply collect the water run-off for a trained analyst to sample and analyze.

As discussed above, a different range of compounds are detected and identified using the field- and laboratory-based water sampling and analysis methods, and as such, the concurrent use of both techniques is necessary to provide for an extensive overview of the hazardous organics present within each water sample. However, the portable GC-MS system can provide rapid field-based intelligence that can be used to reduce the environmental impact of fires and triage the appropriate collection of samples for laboratory analysis.

## Conclusion

Environmental monitoring and management plays an important part in the protection of human and environmental health. The existing monitoring protocols require lengthy and complex sampling and analytical methods where final reporting of results does not occur until some time after the initial pollution event. Due to these delays, results do not play an active role in emergency response scenarios where an immediate response is required by first attending officers. Through the implementation of rapid, on-site sampling and analysis methods, it becomes possible for real-time data to be obtained at the time and location of the pollution event providing actionable scientific data for proactive protection strategies. The field-based water sampling and analysis methods were benchmarked against benchtop instrumentation and conventional laboratory-based methods. The field-based method was capable of detecting and identifying a broad range of hazardous organic compounds in fire water run-off. The SPME sampling method was demonstrated to be capable of extracting a large number of analytes from the samples despite some chromatographic resolution limitations of the portable GC-MS. The field- and laboratory-based methods were able to detect and identify a different range of organic compounds, with few compounds detected simultaneously by both methods. It was therefore determined that these methods can be used in a complementary fashion for an increased understanding of the complete range of hazardous organic compounds that are potentially present in water run-off from a fire scene. Using these techniques, it is possible to achieve real-time sampling and analysis of high concentration contaminants within run-off water to inform emergency response procedures. Through evidence-based decision making using data generated *in-situ*, first responders will be better placed for accurate and timely environmental and human health risk management and mitigation procedures.
